# On a Solution of Iodine in Oil

**Published:** 1849-12

**Authors:** 


*On a Solution of Iodine in Oil.* By M. Marchal.—This preparation has superseded the other forms of iodine at the Val-de-Grâce. M. Marchal, believing that cod-liver oil owes its virtues to the small quantity of iodine which it contains, concluded that a more effective preparation of this substance than the iodide of potassium is found to be, might be made by combining it with an organic body; in which state the drug would probably be longer retained in the economy. He selected an oily body, in the hope that the oil, by forming an emulsion with the bile, would allow of the substance being digested in the small intestines, and enable the stomach to become relieved of its presence. In this way, large doses of iodine can be administered, if requisite, without irritating the latter organ; while the iodine is eliminated by the urine more slowly than is the case with the iodide. At the same time, its absorption is made certain by the fact of its not being detected in the feces. The iodine is dissolved in fresh almond oil as wanted, in the proportion of one part to fifteen, and of this an emulsion is made with gum tragacanth and the milk of almonds. The minimum dose is one grain, gradually increased to six grains. M. Marchal has used it extensively in the treatment of buboes and other glandular enlargements, with the best effects, in promoting and hastening their cure ; M. Ricord also adds his testimony in favor of this preparation.—*Gazette de Hôpitaux.*

